# Exposure of broiler chickens to deoxynivalenol and *Campylobacter jejuni* induces substantial changes in intestinal gene expression

**DOI:** 10.1038/s41598-025-97672-2

**Published:** 2025-04-19

**Authors:** Wageha A. Awad, Daniel Ruhnau, Barbara Doupovec, Claudia Hess, Dian Schatzmayr, Michael Hess, Bertrand Grenier

**Affiliations:** 1 Clinical Centre for Population Medicine in Fish, Pig and Poultry, Clinical Department for Farm Animals and Food System Science, University of Veterinary Medicine, Veterinärplatz 1, 1210 Vienna, Austria; 2 DSM Animal Nutrition and Health, Research Center Tulln, Technopark 1, Tulln, Austria; 3 Current address: LVA GmbH, Klosterneuburg, Austria

**Keywords:** Deoxynivalenol (DON), *Campylobacter jejuni*, Intestinal barrier, Intestinal immune response, Broiler chickens, Microbiology, Bacteria, Bacterial host response

## Abstract

The mycotoxin deoxynivalenol (DON) is of high importance among feed contaminants because of its frequent occurrence in toxicologically relevant concentrations worldwide. Cereal crops, the main component of chicken diet, are commonly contaminated with DON, resulting in frequent exposure of chickens to DON. Likewise, *Campylobacter* (*C.*), a pathogen of major public and animal health concern, is frequently found in chicken flocks and poses a threat to the One Health approach. *Campylobacter* colonizes the gastrointestinal (GI) tract of poultry with a high bacterial load in the caeca. However, the mechanism of *C. jejuni* colonization in chickens is still not understood albeit it is well known that *C. jejuni* resides primarily in the mucosal layer of the chicken intestine. Therefore, in the actual study we focused on the effect of exposure to DON and/or *C. jejuni* on expression profiles of intestinal mucins (MUC1, MUC2), β-defensins (Gallinacin (GAL) 10, 12), cytokines (Toll-like receptor 2 (TLR2), Interleukin (IL) 6, 8, Interferon-γ (IFN)-γ), inducible nitric oxide synthase 2 (iNOS2), as well as selected tight junction proteins (Claudin 5 (CLDN5), Occludin (OCLN), and zonula occludens-1 (ZO1) via RT-qPCR. For this, a total of 150 one-day-old Ross 308 broiler chickens were randomly allocated to six different groups (n = 25 with 5 replicates/group) and were fed for 5 weeks with either contaminated diets (5 or 10 mg DON/kg feed) or basal diets (control). Following oral infection of birds with *C. jejuni* NCTC 12744 at 14 days of age, several changes in gene expression patterns were demonstrated. A significant (*P* ≤ 0.05) downregulation of MUC2 mRNA expression was observed in birds fed DON5 and DON10 diet, as well as in birds co-exposed to DON5 and *C. jejuni* at 7 dpi. Furthermore, at 14 dpi, MUC2 mRNA expression was significantly (*P* ≤ 0.05) downregulated in birds fed DON (5 mg and 10 mg/kg diet) with and without *C. jejuni* and in birds infected solely with *C. jejuni*. The actual study also demonstrated that co-exposure of broiler chickens to DON and *C. jejuni* resulted in a decreased barrier function via downregulation of OCLD mRNA expression. In addition, *Campylobacter* infection induced an increased expression of the antimicrobial peptide GAL12 and the IL8 gene, indicating that *C. jejuni* can initiate an immune response in the chicken gut in a proinflammatory manner. Similarly, DON with and without *C. jejuni* induced upregulation of GAL10 and GAL12 mRNA expression at 7 dpi. Moreover, no change in iNOS2 mRNA expression was observed in both the jejunum and the cecum at either 7 dpi or 14 dpi, suggesting unchanged NO production during exposure/infection. In conclusion, we confirmed that DON contamination corresponding to the currently applicable EU guidance value of 5 mg DON/kg feed affects the intestinal gene expression profiles of broilers, mainly in a dose-independent manner*.* Furthermore, DON exposure interacted synergistically with *C. jejuni* challenge regarding mucins, innate immunity gene expression in either the jejunum or the cecum, suggesting immunomodulatory activity of both foodborne agents (DON and *C. jejuni*).

## Introduction

Gut integrity is crucial for maintaining the physical barrier between the intestinal lumen and the body in order to prevent the adverse impact and the dissemination of pathogens^1–5^. The gut barrier consists of a mucus layer, intestinal epithelial cells, tight junctions (TJ), and the lamina propria. The mucus layer is divided into an outer and an inner layer, which is characterized by high concentration of immunoglobulin A (IgA) and mucin^6^. Mucins are the first line of defence against luminal threats, including toxins, and pathogens^7–9^. It also serves as binding site and nutrient source for bacteria and plays a role in the immunological barrier between the organism and the environment^10–12^. In addition to the mucus layer, TJs, which are not only connecting cells but also form channels that allow permeation between cells, represent an effective barrier against the paracellular penetration of noxious substances and antigens, including bacteria, bacterial toxins, digestive enzymes, and degraded food products^13–15^. Similarly, cytokines also play a crucial role in modulating the intestinal barrier^1,16,17^.

The contamination of food and feed with mycotoxins constitutes a worldwide problem, possessing a potential risk for both animal and human health with significant economic and international trade implications^18^. Deoxynivalenol (DON) is the most common trichothecene mycotoxin detected in feedstuffs worldwide. In chickens, DON affects the epithelial cells of the gastrointestinal tract and contributes to the loss of the epithelial barrier function^19–25^. By inhibition of protein synthesis, which primarily affects rapidly dividing cells, DON can increase the susceptibility to diseases^26–29^.

Likewise, *Campylobacter jejuni* remains the most prevalent foodborne pathogen worldwide causing human gastroenteritis primarily associated with poultry meat. This pathogen remains a major concern for the poultry industry, as clinically asymptomatic carriage of *C. jejuni* is highly prevalent in broiler flocks^30^. In humans, a high prevalence of campylobacteriosis together with an increasing level of antimicrobial resistance, became a major public health concern^31–33^. Recently, in various studies, we have demonstrated the deleterious effects of *C. jejuni* on the chicken intestine^34–36^. To date, there are no data investigating the simultaneous exposure of broilers to DON and *C. jejuni,* although both are widely distributed. We recently demonstrated that DON not only increases the *C. jejuni* load in the intestine of infected birds, but also promotes the translocation of higher numbers of these bacteria to internal organs such as liver and spleen^19,20^. In this context, we found that the co-exposure of broiler chickens to DON and *C. jejuni* affected intestinal paracellular permeability by altering tight junction proteins^17^. Understanding how *C. jejuni* establishes successful colonization in chickens remains a foremost research priority as this gastrointestinal pathogen not only overcomes the host’s defense system, but also competes with the microbial community for space and nutrients. Therefore, further research on the pathogenesis of *C. jejuni* infections in chickens is needed.

Lately, we found that the co-exposure to DON and *C. jejuni* increased intestinal permeability by altering the expression of tight junction proteins^17^. Therefore, it was hypothesized that that the co-exposure to DON and *C. jejuni* could also trigger alterations in the physical and immunological intestinal barrier. *Campylobacter* resides mainly within the mucus of the intestinal tract without causing disease, so the interaction with the mucus layer is of great interest. In in vitro studies, the exposure of *C. jejuni* to mucin (MUC2) has been shown to affect the expression of bacterial genes related to virulence, putative mucin-degrading enzymes, cell morphology, adhesion, and motility^37,38^. It was assumed that MUC2 may act as a cue for the bacterium to enhance its defences against host antimicrobial factors^37^. Currently, the role of mucins as well as innate and adaptive immune genes in *C. jejuni* infection are still elusive. Therefore, to understand the immunological mechanisms underlying the colonization of the chicken gut by *C. jejuni* and how this is influenced by DON dietary contamination, we determined the expression profiles of the intestinal mucins (MUC1, MUC2), β-defensins (Gallinacin (GAL) 10, 12), cytokines (Toll-like receptor 2 (TLR2), Interleukin (IL) 6, 8, Interferon-γ (IFN-γ), inducible nitric oxide synthase 2 (iNOS2), as well as selected tight junction proteins (Claudin 5 (CLDN5), Occludin (OCLN), and zonula occludens-1 (ZO1) by RT-qPCR.

## Material and methods

### Ethics statement

The animal trial was discussed and approved by the institutional ethics committee of the University of Veterinary Medicine and the Ministry of Research and Science under the license number GZ 68.205/0159-WF/V/3b/2017. All methods were performed in accordance with relevant guidelines/regulations and all husbandry practices were performed with full consideration of animal welfare and in accordance with ARRIVE guidelines and regulations.

### Bacterial strains, media, and growth conditions

*Campylobacter jejuni* reference strain NCTC 12744 was cultivated at 41.5 °C for 48 h under microaerophilic conditions (Genbox microaer, BioMeriéux, Vienna, Austria) on CASA agar (BioMerieux, Vienna, Austria) or modified charcoal-cefaprazone-deoxycholate (mCCD) agar (OXOID, Hampshire, UK).

### Birds, housing, and diets

A total of 150 one-day-old male and female broiler chicks were obtained from a commercial hatchery (Ross-308; Geflügelhof Schulz, Lassnitzhöhe, Austria) and divided into six treatment groups, each with an identical set up (n = 25 birds/5 replicates/group): group 1 (control birds supplemented with basal diet); group 2 (birds supplemented with DON 5 mg/kg feed); group 3 (birds supplemented with DON 10 mg/kg feed); group 4 (birds supplemented with DON 5 mg/kg feed + *C. jejuni*); group 5 (birds supplemented with DON 10 mg/kg feed + *C. jejuni*); group 6 (birds infected with *C. jejuni* and supplemented with the basal diet).

On the day of hatch, chicks were tagged, weighed, and kept in floor pens on wood shavings under strict biosecurity. Light was set at a 16:8 light/dark cycle. Temperature was kept at 35 °C during the first days of life and reduced to 25 °C with the age of birds. Feed and water were provided ad libitum. The birds were fed for 5 consecutive weeks with either contaminated diets with 5 mg or 10 mg DON/kg feed, or basal diets (control, non-contaminated diet during the starter and grower periods). The control diet was prepared with non-contaminated wheat. The DON contaminated diet was prepared by replacing “non-contaminated” control wheat with DON contaminated wheat. The composition of the diet comprised maize, wheat, soy, soybean meal, soybean oil, rapeseed oil and a premix of vitamins, minerals, monocalcium phosphate and salt. Starter diet was fed for 10 days, followed by the grower diet from day 11 until 35 days of age. Representative feed samples for each group were analysed for determining the concentration of DON in the diets.

Cloacal swabs were taken on day one and prior to infection to confirm that birds were free of *Campylobacter*. The swabs were directly streaked onto mCCDA agar, and grown for 48 h under microaerophilic conditions at 41.5 °C. On day 14 of age, chicks from groups 4, 5 and 6 were orally inoculated into the crop via a feeding tube with a dose of 1 × 10^8^ CFU/ml/bird as previously described^34^. Until the end of the animal trial, the birds were monitored daily for any adverse effects or clinical signs. The effect of DON and/or *C. jejuni* on intestinal permeability and bacterial translocation are published in a separate paper^17,25^.

At 7- and 14-days post infection (dpi), five birds from each group were randomly selected (1/replicate) and euthanized by injection of a single dose of thiopental (20 mg/kg) into the wing vein and killed by bleeding of the jugular vein. For RT-qPCR, samples from jejunum and cecum were taken, washed with ice-cold buffered saline, placed in RNA later and stored at − 80 °C until further use. Furthermore, *C. jejuni* in jejunum and cecum were enumerated by preparing tenfold dilutions in phosphate-buffered saline with a PH of 7.4 (PBS, Thermo Fisher Scientific Inc., Gibco Life Technologies Corporation) and plated on CASA agarin duplicate, followed by microaerobic incubation at 41.5 °C for 48 h.

### Gene expression analysis of selected tight junctions, mucins, and cytokines by real-time qPCR

Approximately 30 mg of the intestinal samples (jejunum and cecum) were homogenized via a bead-beating step, and the total RNA was extracted using the RNeasy Mini Kit (Qiagen, Hilden, Germany) according to manufacturer’s instruction (including the DNase I treatment). The concentration and purity of RNA were measured using A260/280 and A260/230 ratios by Nanodrop2000 spectrophotometry (Thermo Fisher Scientific, Vienna, Austria), and RNA integrity (RIN) was analyzed for all samples with the Bioanalyzer 2100 (Agilent Technologies, Waldbronn, Germany). The average RIN was > 9.0 for all the RNA extracted.

Isolated RNA samples were shipped on dry ice to Qiagen GmbH (Hilden, Germany), where cDNA synthesis, and RT-qPCR was conducted. The threshold cycle (Ct) values for all genes were provided by Qiagen and used for data analysis. First, the ∆Ct (normalized Ct) value for each sample was calculated by subtracting the Ct value for the target gene from the mean Ct value of the two housekeeping genes, and then converted to the individual expression 2^−(∆Ct)^^39^. This value was subsequently used for statistical evaluation and expressing the fold change. Twelve genes were selected for evaluating the effect of the treatments on their expression, and were as follows: MUC1, MUC2, CLDN5, OCLN, ZO1, GAL10, GAL12, TLR2, IL6, IL8, IFN-γ, iNOS2. Glyceraldehyde 3-phosphate dehydrogenase (GAPDH) and ribosomal protein L4 (RPL4) were used as housekeeping genes for normalization.

### Statistical analysis

GraphPad Prism was used to run the statistical analysis on the gene expression. A 2-way ANOVA factorial design, with Challenge and DON (either 5 or 10 ppm) as the two factors, was used to evaluate the type of interaction (i.e. antagonistic, additive, or synergistic), and followed by Dunnett’s multiple comparison test for pairwise comparison (to the negative control group, no challenge and 0 ppm DON).

## Results

The initial body weight (BW) on day 1 did not differ (*P* ≥ 0.05) between groups. At the end of the trial (d 35), a significant decreased in BW (*P* ≤ 0.05) was found in birds fed with DON contaminated diets with or without *C. jejuni* compared to the control group. At d 35, the body weight was significantly (*P* = 0.030) lower (1641 ± 89 g) in the DON10 group compared with the control group (1878 ± 73 g). Likewise, birds in *C. jejuni*-infected groups had significantly lower body weight (*P* ≤ 0.05) than birds in non-infected groups. Moreover, the overall body weight gain was significantly reduced in the DON10 group with (1514 ± 58 g; *P* = 0.005) and without *C. jejuni* (1590 ± 88 g; *P* = 0.035) compared with the control group (1832 ± 73 g).

In birds inoculated with *C. jejuni* the bacteria were detected in the jejunum, cecum, liver and spleen at all time points as analysed by bacterial plating. In birds fed DON10-contaminated diets, a trend towards increased *Campylobacter* loads was noted in the jejunum (0.43 × 10^5^ ± 0.30 CFU/g) and in the cecum (2.58 × 10^11^ ± 0.96 CFU/g) at 21 dpi. Furthermore, feeding of DON resulted in an increase in *Campylobacter* translocation to the spleen (2.00 × 10^3^ ± 0.16 CFU/g) at 7 dpi (*P* < 0.001) and to liver (1.73 × 10^2^ ± 0.35 CFU/g) at 14 dpi.

Changes in the expression of intestinal mucins (MUC1, MUC2), tight junction proteins (CLDN5, OCLN, and ZO1), antimicrobial peptides β-defensins (GAL10, 12), cytokines (TLR2, IL6, IL8, IFN-γ), and iNOS2 in both jejunum and cecum of either control or infected birds with or without DON are shown in Tables 1 and 2.

**Table 1:** Gene expression analysis in the chicken jejunum.

Genes	DON treatment	7 days post challenge						14 days post challenge					
		No challenge			Challenge with *C. jejuni*			No challenge			Challenge with *C. jejuni*		
		0 ppm	5 ppm	10 ppm	0 ppm	5 ppm	10 ppm	0 ppm	5 ppm	10 ppm	0 ppm	5 ppm	10 ppm
MUC1	Fold change	1.00	1.08	3.00	0.58	1.26	0.37	1.00	4.41	1.94	2.09	0.82	1.39
	Stand. dev.	0.84	0.90	2.30	0.57	0.69	0.05	0.64	3.48	1.61	1.63	0.83	1.76
	Fold regulation		1.08	3.00	− 1.74	1.26	− 2.67		4.41*	1.94	2.09	− 1.22	1.39
MUC2	Fold change	1.00	1.44	0.89	0.25	0.70	0.99	1.00	0.67	0.95	0.62	1.35	1.17
	Stand. dev.	0.36	0.47	0.18	0.38	0.63	0.24	0.71	0.62	0.69	0.77	0.26	0.29
	Fold regulation		1.44	− 1.12	**− 4.04**	− 1.43	− 1.01		− 1.50	− 1.06	− 1.61	1.35	1.17
CLDN5	Fold change	1.00	1.40	1.27	0.97	1.15	1.04	1.00	1.23	1.01	1.04	1.08	1.09
	Stand. dev.	0.21	0.65	0.45	0.09	0.55	0.24	0.22	0.18	0.14	0.35	0.27	0.34
	Fold regulation		1.40	1.27	− 1.04	1.15	1.04		1.23	1.01	1.04	1.08	1.09
OCLN	Fold change	1.00	1.33	1.50	0.77	1.25	1.34	1.00	0.92	1.13	0.84	1.27	1.18
	Stand. dev.	0.40	0.24	0.59	0.57	0.60	0.43	0.25	0.35	0.31	0.66	0.23	0.14
	Fold regulation		1.33	1.50	− 1.29	1.25	1.34		− 1.08	1.13	− 1.18	1.27	1.18
ZO1	Fold change	1.00	1.55	1.16	1.05	1.13	1.47	1.00	0.72	0.71	0.75	0.82	0.75
	Stand. dev.	0.21	0.49	0.44	0.15	0.34	0.36	0.12	0.10	0.14	0.22	0.14	0.10
	Fold regulation		**1.55**	1.16	1.05	1.13	1.47		**− 1.39**	**− 1.40**	**− 1.34**	**− 1.22**	**− 1.34**
GAL10	Fold change	1.00	3.53	8.77	0.92	1.35	1.23	1.00	0.74	1.06	0.29	0.84	0.92
	Stand. dev.	0.37	4.14	15.58	0.61	0.77	0.89	0.72	0.57	1.18	0.11	0.93	0.74
	Fold regulation		3.53	8.77	− 1.08	1.35	1.23		− 1.36	1.06	− 3.41	− 1.19	− 1.09
GAL12	Fold change	1.00	0.56	0.57	2.12	1.20	0.92	1.00	0.58	0.52	0.65	0.13	0.09
	Stand. dev.	0.78	0.27	0.35	1.16	0.90	0.76	0.94	0.84	0.68	0.44	0.14	0.04
	Fold regulation		− 1.79	− 1.75	2.12	1.20	− 1.09		− 1.73	− 1.92	− 1.54	− 7.66	− 11.14
TLR2	Fold change	1.00	0.59	1.33	0.83	0.96	1.59	1.00	1.19	1.11	1.47	0.74	1.32
	Stand. dev.	0.64	0.35	0.65	0.58	0.22	1.17	0.92	0.93	0.74	0.99	0.22	0.55
	Fold regulation		− 1.69	1.33	− 1.20	− 1.04	1.59		1.19	1.11	1.47	− 1.35	1.32
IL6	Fold change	1.00	1.30	0.88	0.74	1.11	0.85	1.00	2.29	1.45	2.94	1.61	2.85
	Stand. dev.	0.52	0.54	0.24	0.22	0.53	0.23	0.32	2.10	0.72	1.59	0.82	2.36
	Fold regulation		1.30	− 1.14	− 1.36	1.11	− 1.18		2.29	1.45	2.94	1.61	2.85
IL8	Fold change	1.00	1.36	0.34	1.02	2.24	0.31	1.00	0.45	0.41	0.48	0.18	0.64
	Stand. dev.	1.10	1.69	0.43	0.79	2.88	0.16	1.50	0.33	0.47	0.37	0.05	0.81
	Fold regulation		1.36	− 2.90	1.02	2.24	− 3.20		− 2.25	− 2.45	− 2.07	− 5.62	− 1.57
IFN-γ	Fold change	1.00	1.15	0.71	0.53	0.58	0.98	1.00	0.68	0.66	0.95	0.56	0.95
	Stand. dev.	0.88	1.63	0.36	0.59	0.57	0.79	0.60	0.52	0.24	0.55	0.36	0.59
	Fold regulation		1.15	− 1.41	− 1.88	− 1.72	− 1.02		− 1.47	− 1.52	− 1.06	− 1.79	− 1.06
iNOS2	Fold change	1.00	1.04	1.16	1.10	0.97	1.18	1.00	0.69	0.68	0.73	0.76	0.67
	Stand. dev.	0.26	0.44	0.29	0.45	0.59	0.33	0.20	0.09	0.09	0.25	0.13	0.11
	Fold regulation		1.04	1.16	1.10	− 1.03	1.18		**− 1.45**	**− 1.47**	− 1.37	− 1.31	**− 1.49**

*Cells highlighted in bold indicate a significant difference with the negative control at that time point (i.e. no challenge and DON 0 ppm; Dunnett’s multiple comparison test).

**Table 2:** Gene expression analysis in the chicken cecum.

Genes	DON treatment	7 days post challenge						14 days post challenge					
		No challenge			Challenge with *C. jejuni*			No challenge			Challenge with *C. jejuni*		
		0 ppm	5 ppm	10 ppm	0 ppm	5 ppm	10 ppm	0 ppm	5 ppm	10 ppm	0 ppm	5 ppm	10 ppm
MUC1	Fold change	1.00	1.13	1.14	0.44	1.47	1.00	1.00	0.39	0.84	0.56	0.45	0.45
	Stand. dev.	1.26	1.26	1.55	0.28	1.04	1.38	0.94	0.33	0.62	0.46	0.36	0.35
	Fold regulation		1.13	1.14	− 2.29	1.47	− 1.00		− 2.57	− 1.19	− 1.80	− 2.21	− 2.21
MUC2	Fold change	1.00	0.05	0.06	1.06	0.28	1.06	1.00	0.05	0.08	0.07	0.12	0.11
	Stand. dev.	0.85	0.04	0.06	0.99	0.48	0.64	0.90	0.05	0.05	0.04	0.11	0.11
	Fold regulation		− 18.75	− 15.97	1.06	− 3.58	1.06		− 18.76*	**− 12.61**	**− 14.70**	**− 8.22**	**− 9.45**
CLDN5	Fold change	1.00	0.94	1.10	1.11	0.88	1.26	1.00	0.76	0.70	1.00	0.72	0.82
	Stand. dev.	0.22	0.49	0.31	0.71	0.34	0.26	0.10	0.28	0.12	0.64	0.37	0.34
	Fold regulation		− 1.07	1.10	1.11	− 1.13	1.26		− 1.32	− 1.43	− 1.00	− 1.38	− 1.21
OCLN	Fold change	1.00	0.60	0.66	1.36	0.57	1.23	1.00	0.61	0.41	0.53	0.60	0.67
	Stand. dev.	0.33	0.26	0.22	0.99	0.38	0.15	0.41	0.36	0.09	0.39	0.27	0.45
	Fold regulation		− 1.66	− 1.52	1.36	− 1.75	1.23		− 1.64	− 2.44	− 1.87	− 1.65	− 1.49
ZO1	Fold change	1.00	0.98	1.07	1.20	0.86	1.10	1.00	1.03	0.84	0.77	0.99	0.92
	Stand. dev.	0.18	0.04	0.19	0.74	0.19	0.19	0.12	0.53	0.27	0.48	0.24	0.18
	Fold regulation		− 1.02	1.07	1.20	− 1.16	1.10		1.03	− 1.18	− 1.30	− 1.01	− 1.08
GAL10	Fold change	1.00	1.03	1.59	3.56	13.04	2.06	1.00	0.18	0.22	0.37	0.94	0.20
	Stand. dev.	0.90	0.73	1.10	6.19	22.10	0.32	0.68	0.08	0.17	0.30	1.26	0.07
	Fold regulation		1.03	1.59	3.56	13.04	2.06		− 5.53	**− 4.57**	**− 2.72**	− 1.06	**− 5.01**
GAL12	Fold change	1.00	5.64	3.83	1.19	2.85	1.59	1.00	4.34	3.68	3.18	2.76	3.19
	Stand. dev.	1.57	5.40	1.84	1.02	1.28	1.75	0.85	4.15	2.11	2.74	1.53	2.23
	Fold regulation		5.64	**3.83**	1.19	2.85	1.59		4.34	3.68	3.18	2.76	3.19
TLR2	Fold change	1.00	2.57	2.40	1.07	1.41	1.11	1.00	0.66	1.14	1.22	1.27	1.40
	Stand. dev.	0.49	1.98	2.17	0.53	0.70	0.55	0.29	0.25	0.57	0.69	0.92	0.60
	Fold regulation		2.57	2.40	1.07	1.41	1.11		− 1.50	1.14	1.22	1.27	1.40
IL6	Fold change	1.00	2.34	1.01	0.79	1.20	2.01	1.00	0.83	1.66	1.58	1.54	1.41
	Stand. dev.	0.77	2.74	0.45	0.25	0.85	1.65	0.70	0.30	0.65	1.07	1.02	1.46
	Fold regulation		2.34	1.01	− 1.26	1.20	2.01		− 1.21	1.66	1.58	1.54	1.41
IL8	Fold change	1.00	6.04	5.78	1.45	4.16	0.72	1.00	1.52	2.26	4.23	1.56	2.33
	Stand. dev.	0.70	9.40	3.94	1.56	5.25	0.54	0.91	0.78	3.17	3.18	1.42	1.46
	Fold regulation		6.04	**5.78**	1.45	4.16	− 1.38		1.52	2.26	**4.23**	1.56	2.33
IFN-γ	Fold change	1.00	2.34	0.91	1.34	1.06	0.63	1.00	0.95	2.20	2.06	1.38	1.28
	Stand. dev.	0.46	2.92	0.42	1.23	0.70	0.60	0.36	0.31	2.38	1.35	0.84	0.45
	Fold regulation		2.34	− 1.09	1.34	1.06	− 1.58		− 1.05	2.20	2.06	1.38	1.28
iNOS2	Fold change	1.00	6.15	1.56	1.16	1.04	1.22	1.00	0.68	0.97	1.39	0.78	1.03
	Stand. dev.	0.21	11.37	1.30	0.60	0.31	0.38	0.23	0.22	0.76	1.35	0.31	0.53
	Fold regulation		6.15	1.56	1.16	1.04	1.22		− 1.46	− 1.04	1.39	− 1.29	1.03

*Cells highlighted in bold indicate a significant difference with the negative control at that time point (i.e. no challenge and DON 0 ppm; Dunnett’s multiple comparison test).

### Barrier forming mucins and tight junctions’ mRNA expression in jejunum and cecum

**Fig. 1:**
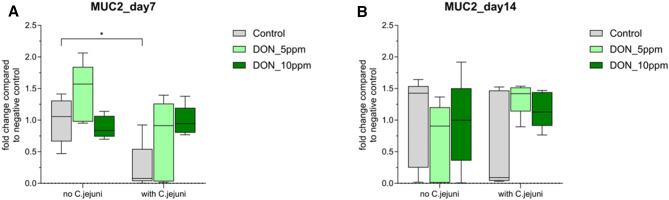
Mucin 2 mRNA expression levels in jejunum (**a**) day7 post challenge (**b**) day14 post challenge measured by reverse transcription‐qPCR. The expression level is shown as fold change of mRNA expression levels (n = 5, *P* ≤ 0.05); 2-way ANOVA with Tukey’s multiple comparisons test.

In the jejunum, MUC1 mRNA expression was upregulated in birds fed with DON10 and in *C. jejuni* infected birds at 7 dpi but, this effect didn’t reach statistical significance (Table 1). On the contrary, MUC1 mRNA expression tended to be downregulated in birds co-exposed to DON10 and *C. jejuni* at 7 dpi. Later, at 14 dpi, MUC1 mRNA expression was significantly increased in birds fed DON5 (*P* ≤ 0.05) (Table 1). While *C. jejuni* alone significantly (*P* ≤ 0.05) reduced the expression of MUC2 at 7 dpi, the co-exposure to DON and *C. jejuni* significantly (*P* ≤ 0.05) upregulated this mRNA expression (Fig. 1).

**Fig. 2:**
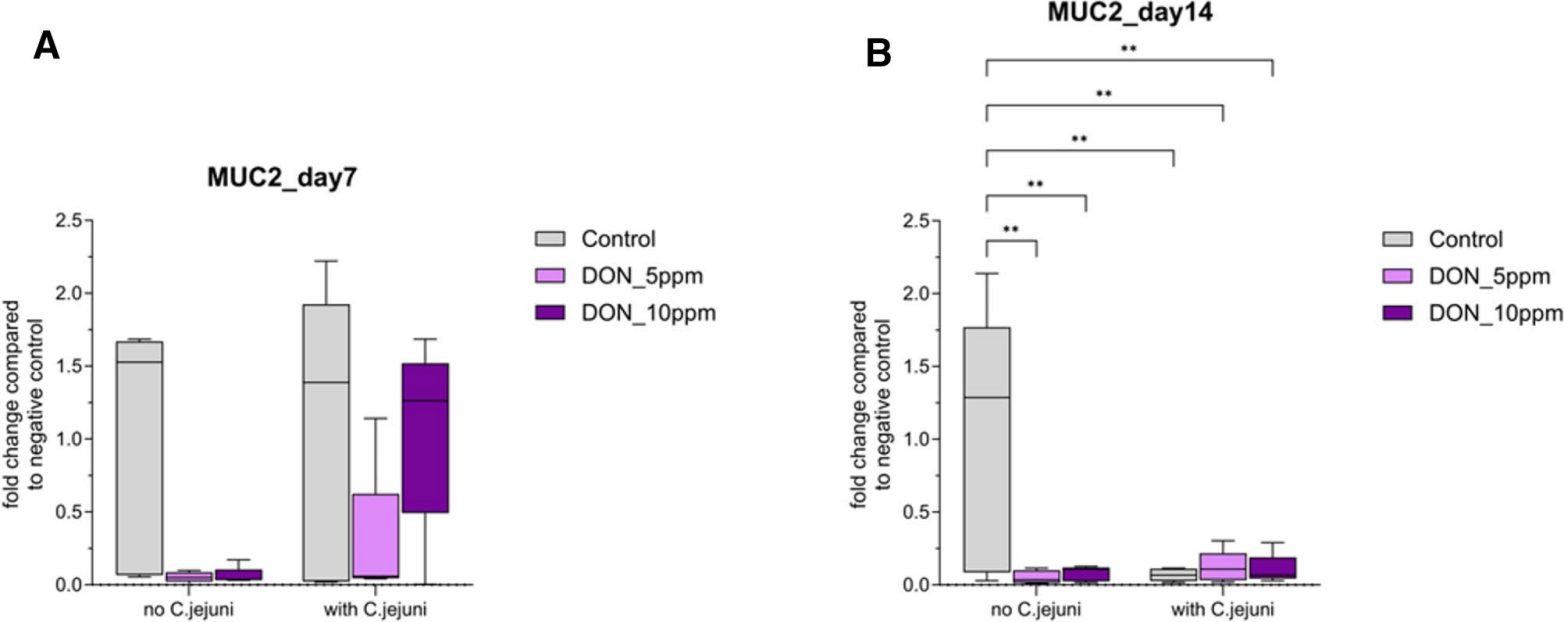
Mucin 2 mRNA expression levels in cecum (**a**) day7 post challenge (**b**) day14 post challenge measured by reverse transcription‐qPCR. The expression level is shown as fold change of mRNA expression levels (n = 5, *P* ≤ 0.05); 2-way ANOVA with Tukey’s multiple comparisons test.

Likewise, in the cecum, co-exposure to DON5 and *C. jejuni* resulted in upregulation of MUC1 mRNA expression at 7 dpi, but the effect didn’t reach statistical significance (*P* ≥ 0.05, Table 2). On the contrary, at 14 dpi, the MUC1 mRNA expression was significantly (*P* ≤ 0.05) downregulated in DON5 and all infected birds with or without DON (Table 2). Furthermore, a significant (*P* ≤ 0.05) downregulation of MUC2 mRNA expression was observed in birds fed DON5 and DON10 diet, as well as in birds co-exposed to DON5 and *C. jejuni* at 7 dpi. Similarly, MUC2 mRNA expression was significantly (*P* ≤ 0.05) downregulated in birds fed DON5 and DON10 diets with and without *C. jejuni* and in birds infected with *C. jejuni* alone at 14 dpi (Fig. 2).

Furthermore, feeding of DON with and without *C. jejuni* didn’t alter the mRNA expression of CLDN5 and ZO1 in either the jejunum or the cecum at 7 and 14 dpi (Tables 1 and 2), indicating non-significant changes in barrier-forming claudin (CLDN5) and the cytosolic tight junction (ZO1). However, OCLD mRNA expression was significantly reduced at 14 dpi only in birds fed DON10 and in birds infected with *C. jejuni*.

### Expression of β-defensins and cytokines mRNA in jejunum and cecum

**Fig. 3:**
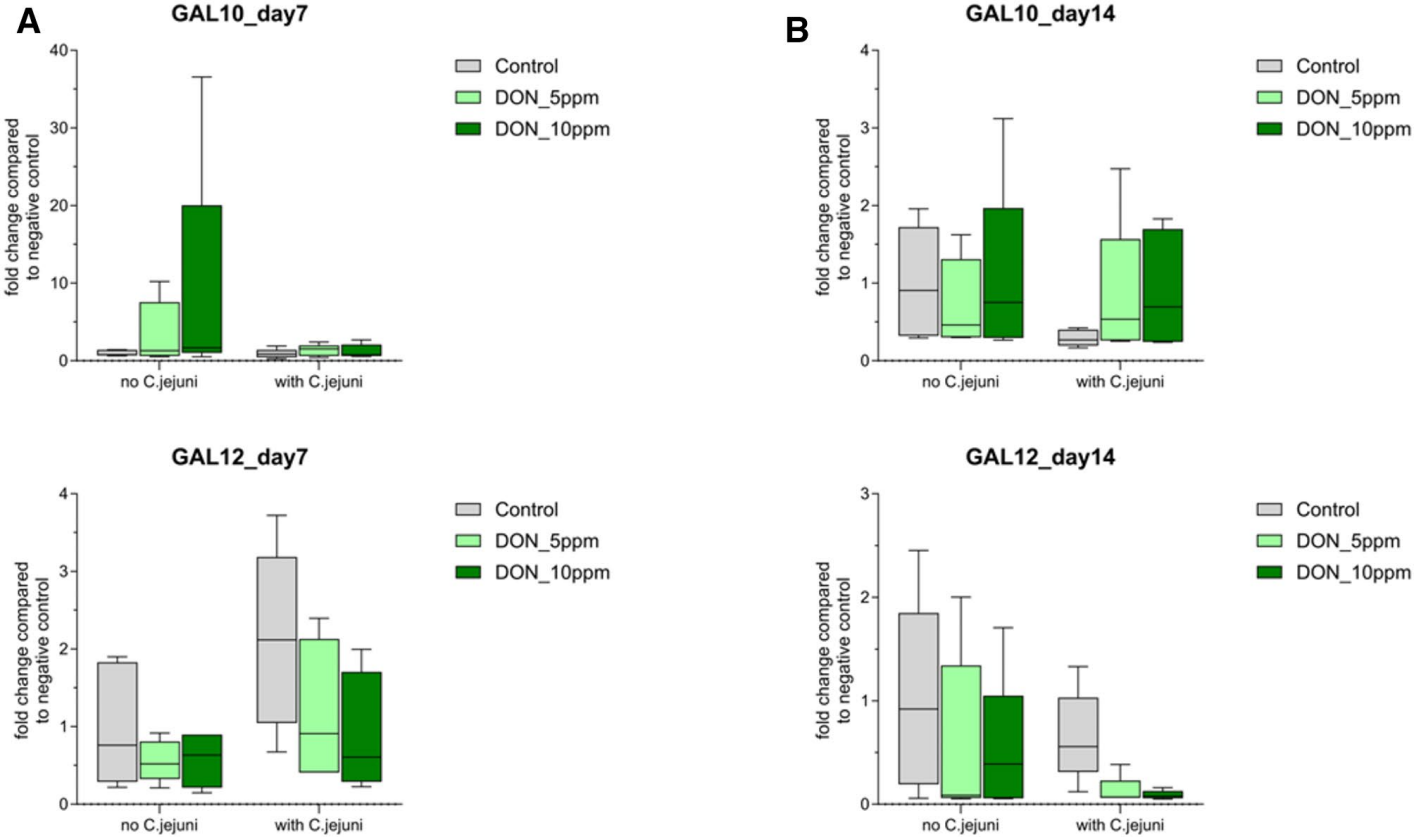
Gallinacin 10 and Gallinacin 12 mRNA expression levels in jejunum (**a**) day7 post challenge (**b**) day14 post challenge measured by reverse transcription‐qPCR. The expression level is shown as fold change of mRNA expression levels (n = 5, *P* ≤ 0.05); 2-way ANOVA with Tukey’s multiple comparisons test.

In the jejunum, the mRNA expression of GAL10 at 7 dpi was mainly affected by DON feeding, while infection with *C. jejuni* had no effect on the expression of this protein (Table 1). However, *C. jejuni* increased GAL12 expression at 7 dpi (Table 1; Fig. 3). At 14 dpi, *C. jejuni* reduced the expression of GAL10, and DON with and without *C. jejuni* reduced the expression of GAL12 (*P* ≤ 0.05).

**Fig. 4:**
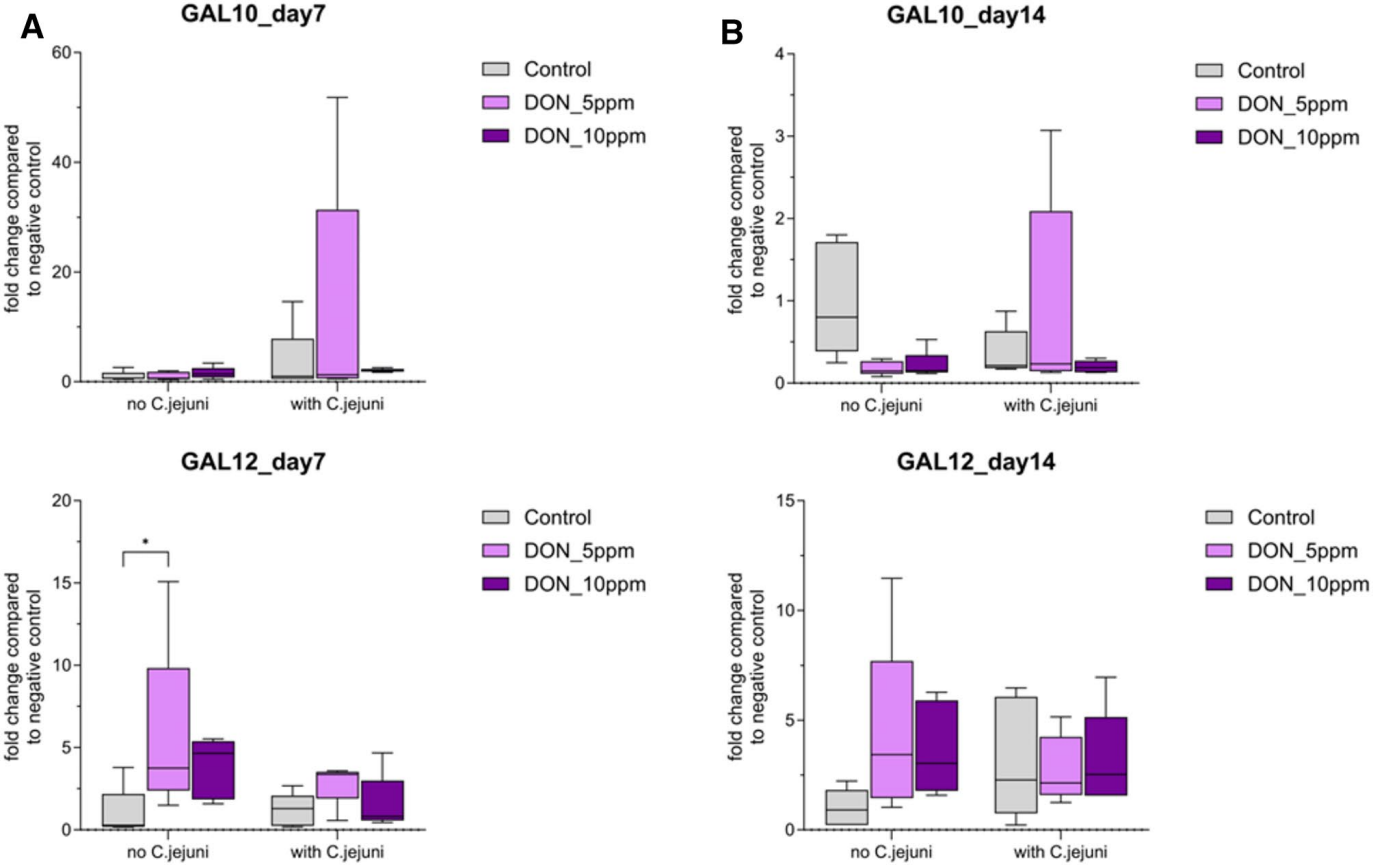
Gallinacin 10 and Gallinacin 12 mRNA expression levels in cecum (**a**) day7 post challenge (**b**) day14 post challenge measured by reverse transcription‐qPCR. The expression level is shown as fold change of mRNA expression levels (n = 5, *P* ≤ 0.05); 2-way ANOVA with Tukey’s multiple comparisons test.

In the cecum, the co-exposure to DON and *C. jejuni* upregulated the mRNA expression of GAL10 (*P* ≤ 0.05) at 7 dpi*.* Furthermore, DON with and without *C. jejuni* increased (*P* ≤ 0.05) the expression of GAL12 (Fig. 4)*.* Later, at 14 dpi, the expression of GAL10 was reduced in all groups (Table 1). On the contrary, both DON and *C. jejuni* increased the expression of GAL12 at 14 dpi (Fig. 4).

In the jejunum, IL8 mRNA expression was significantly (*P* ≤ 0.05) downregulated in chickens fed a higher DON10 concentration at 7 dpi, providing evidence that higher DON exposure decreased IL8 transcription. In contrast, *C. jejuni* alone increased the expression of IL-8, but this effect didn’t reach statistical significance (*P* ≥ 0.05). Nevertheless, IL8 mRNA expression was downregulated at 14 dpi in all groups, with significant values in chickens co-exposed to lower levels of DON5 and *C. jejuni* (Table 1).

In the cecum, both lower and higher DON concentration significantly (*P* ≤ 0.05) upregulated IL8 mRNA expression at 7 dpi, while *C. jejuni* significantly (*P* ≤ 0.05) upregulated IL8 mRNA expression at 14 dpi (Table 1).

The results also showed that there were no significant differences in IFN-γ mRNA expression in the jejunum or cecum at either 7 dpi or 14 dpi, and the expression was only upregulated at 7 dpi in birds, co-exposed to DON10 and *C. jejuni* (Tables 1 and 2).

In parallel, no change in iNOS2 mRNA expression was observed in the jejunum or cecum at 7 dpi and 14 dpi (Tables 1 and 2), indicating unchanged NO production during infection.

Finally, DON exposure interacted synergistically with *C. jejuni* challenge regarding mucins, innate immunity and gene expression in either the jejunum or the cecum (Tables 1 and 2), suggesting immunomodulatory activity of both foodborne agents (DON and *C. jejuni*). However, in chickens fed with DON10 and infected with *C. jejuni,* a significant antagonist effect was observed in the jejunum at 7 dpi and in the cecum at 14 dpi (*P* ≤ 0.05). The results are useful in predicting the effects of additively acting noxes agents (two widespread foodborne agents) and in visualizing the magnitude of additive effects in accordance with the results of two-way analysis of variances.

## Discussion

The mycotoxin deoxynivalenol (DON) is of great health concern in poultry production. DON affects the epithelial cells of the gastrointestinal tract and contributes to the loss of the epithelial barrier function. Likewise, *C. jeuni* remains a major concern for the poultry industry, as the prevalence of *C. jejuni* in European broiler flocks with an average of 71% is well documented^40^. Recently, we were able to show that *C. jejuni*, contrary to the general belief, increases intestinal permeability or “leaky gut” and promotes not only the translocation of *C. jejuni* itself but also the spread of *Escherichia coli* to internal organs^19,35,41^. Consequently, bacterial invasiveness is part of the colonization strategy that leads to persistent infection in chickens^42^. However, there is a certain variation between *C. jejuni* strains in terms of both extra-intestinal spread and colonization of the gastrointestinal tract^43^. In addition, triggering of the innate and acquired immune response especially in the early phase of colonization contributes to these differences^44^. Recently, we found that the co-exposure to DON and *C. jejuni* increased intestinal permeability by altering the expression of tight junction proteins^17^. Thus, we hypothesized that that the co-exposure of chickens to DON and *C. jejuni* may also induce changes in the physical and immunological intestinal barrier.

In the current study, most differentially expressed genes had low fold-changes following *C. jejuni* inoculation, expect MUC2 in both jejunum and cecum, indicating that MUC2 expression strongly downregulated in *Campylobacter* infection*.* The results can be explained as *C. jejuni* uses mucin as a source of energy^15,45,46^. Furthermore, it was shown in an *in vitro* study that *C. jejuni* utilizes MUC2 as a key for the modulation of expression of various genes related to the pathogenicity of *C. jejuni*^37^. Likewise, DON also reduced the mRNA expression of MUC2. This is in agreement with Antonissen et al.^47^ who reported that the expression of the gene coding for MUC2 was significantly downregulated in the duodenum of broilers fed DON. The actual results showed that either DON or *C. jejuni* can negatively affect the production of intestinal mucus, which plays an important role in gut barrier function as MUC2 is the main component of the mucus layer that covers the intestinal epithelium, thereby fighting bacterial infections and maintaining the integrity of the epithelial barrier^48^.

Van Deun et al.^42^ postulated that high levels of persistent colonization and the absence of pathology associated with *C. jejuni* infection are due to immunomodulatory mechanisms specific to poultry. The innate immune response is a crucial factor of subsequent adaptive immune activation and outcome of infection^49^. Gallinacins 1–13 (GAL 1–13) are antimicrobial peptides that, as components of the innate immunity, regulate immune homeostasis, mucosal barrier function, and intestinal development. They act against a broad spectrum of microorganisms, including gram-positive and gram-negative bacteria, fungi, and yeast^50–52^^,^^53^. Therefore, we examined the effects of DON with and without *C. jejuni* on the mRNA expression of the antimicrobial peptides (GAL10, and GAL12) in both jejunum and cecum of broiler chickens.

The present study showed that DON/or and *C. jejuni* induced upregulation of GAL10 and GAL12 mRNA expression in the jejunum and cecum at 7 dpi, suggesting that the immune system of the chickens reacts straight after infection. On the contrary, at 14 dpi, the expression of GAL10 and GAL12 was downregulated in the jejunum, but the expression of GAL12 increased in the cecum in all birds exposed to DON/or and *C. jejuni,* demonstrating a directed innate immune response against this intestinal pathogen. Likewise, Li et al.^54^ found that *C. jejuni* upregulated AvBD 10 and 12 at 7 dpi in the cecum of chickens inoculated with *C. jejuni* on the first day of age. The results provide evidence for dynamic cross talk between *C. jejuni* and host epithelial innate defence. We also found that DON/or and *C. jejuni* in the cecum upregulated the IL8 mRNA expression during infection, while it was downregulated in the jejunum at 7 dpi and 14 dpi. It was shown that up-regulation of the relative gene expression of IL8 in case of DON toxicity due to the decrease of the protein synthesis in the intestinal cells^55^. Earlier, Hickey et al.^56^ has also shown that various *C. jejuni* strains induce different levels of IL8 secretion, which is directly related to their invasive potential.

In this study, we also found that IFN-γ mRNA expression was upregulated in the cecum during early infection, while it was downregulated in the jejunum in birds co-exposed to DON and *C. jejuni*. Overall, an explanation for the differences in the change between jejunal and cecal gene expression might be due to direct or indirect effects of gut microbiota, as the density of microbes is higher in the cecum than in the jejunum, which plays a crucial role in modulating the gut immune barrier. To further characterize the host defence against *C. jejuni*, we measured the gene expression of iNOS2, which is part of the innate defense mechanisms. In contrast to humans, we found that feeding DON and/or *C. jejuni* did not alter iNOS mRNA expression in either the jejunum or the cecum at any time point, suggesting that *C. jejuni* might not be cleared and is able to colonize the bird without causing clinical signs. It has been shown that mammalian cells upregulate NO production, and that NO plays a significant role in the immune response to this pathogen^57^. It was shown also that NO can be cytostatic for enteroinvasive bacteria and may decrease microbial entry into epithelial cells by increasing the epithelial protective barrier through the release of epithelial mucus^58^.

Taken together, the host´s intestinal mucosal defense against *C. jejuni* is crucial to the outcome of infection. The results also suggest that DON may have an impact on the innate immune response, which may affect the resistance of chickens to infectious diseases and consequently increase the host susceptibility to infection. Furthermore, DON exposure interacted synergistically with *C. jejuni* challenge in terms of mucins, innate immunity gene expression in either the jejunum or the cecum. A possible explanation for the synergistic effect could be that DON treatment induces changes in the intestinal microenvironment by affecting the composition of the microbiota, the production or composition of mucus, or the production of local antimicrobial defenses^19,59^.

In conclusion, we have demonstrated that both DON and *C. jejuni* affected the physical intestinal barrier of broiler chickens in specific ways. Those results are of practical relevance as the current EU guidance value for the tolerable level of DON in poultry diets is 5 mg/kg feed (European Commission No. 1881/2006). Furthermore, the results indicate that gene expression differs in magnitude under various challenging conditions (DON or *C. jejuni)*, which might provide important aspects for the development of specific control mechanisms and thereby reduce contamination levels along the human food chain. Overall, the results of the current study support the hypothesis that DON could influence the infection profile of *C. jejuni*, a subject not proven so far. This in turn leads to greater interest in understanding bacterial responses to DON and the mechanisms involved, to provide a more comprehensive picture of the changes caused by DON in prokaryotes. In addition, the permissible concentrations of DON contamination in feed need to be reconsidered as a potential stress factor for the gut health of broilers.

## Data Availability

The datasets generated during the current study are available from the corresponding author on reasonable request.
